# The effect of amidation on the behaviour of antimicrobial peptides

**DOI:** 10.1007/s00249-015-1094-x

**Published:** 2016-01-08

**Authors:** Manuela Mura, Jianping Wang, Yuhua Zhou, Marco Pinna, Andrei V. Zvelindovsky, Sarah R. Dennison, David A. Phoenix

**Affiliations:** UCLan Biomedical Technology Limited (Shenzhen), Shenzhen Virtual University Park, Shenzhen, 518057 People’s Republic of China; Computational Physics Group, School of Mathematics and Physics, University of Lincoln, Brayford Pool, Lincoln, LN6 7TS UK; School of Pharmacy and Biomedical Science, University of Central Lancashire, Preston, PR1 2HE UK; School of Applied Science, London South Bank University, 103 Borough Road, London, SE1 0AA UK

**Keywords:** Antimicrobial peptides, Membrane, Molecular dynamics, Secondary structure, Cooperative effect, Amino acid, Anticancer

## Abstract

Aurein 2.6-COOH and aurein 3.1-COOH were studied along with their naturally occurring C-terminally amidated analogues. Circular dichroism (CD) and molecular dynamic (MD) simulations were used to study the effects of amidation on the interaction of antimicrobial peptides (AMPs) with lipid bilayers. CD measurements and MD analysis suggested that both peptide analogues were predominantly random coil and adopted low levels of $\alpha$-helical structure in solution (<30 %) and in the presence of a lipid bilayer the peptides formed a stable $\alpha$-helical structure. In general, amidated analogues have a greater propensity than the non-amidated peptides to form a $\alpha$-helical structure. MD simulations predicted that aurein 2.6-COOH and aurein 3.1-CHOOH destabilised lipid bilayers from 1,2-dimyristoyl-sn-glycero-3-phosphocholine and 1,2-dimyristoyl-sn-glycero-3-phosphoserine via angled bilayer penetration. They also showed that aurein 2.6-CONH$_2$ and aurein 3.1-CONH$_2$ formed a helix horizontal to the plane of an asymmetric interface.

**Electronic supplementary material:**

The online version of this article (doi:10.1007/s00249-015-1094-x) contains supplementary material, which is available to authorized users.

## Introduction

Antimicrobial peptides (AMPs) form a part of the innate immune system across eukaryotic organisms and have potent therapeutic activity against a wide spectrum of bacteria and fungi (Li et al. 2012; Brandenburg et al. 2012; Dennison et al. 2005). However, the mechanism of antimicrobial action of these peptides has yet to be fully elucidated and remains the subject of continuous research (Phoenix et al. 2013; Fox 2013; Wimley and Hristova 2011; Yeaman and Yount 2003). It is generally accepted that the ability of AMPs to kill microorganisms depends upon their ability to target the membranes of these organisms (Huang and Huang 2010; Strömstedt et al. 2010). AMPs are usually short molecules of up to 50 amino acid residues and a number have been shown to penetrate the membrane in an oblique orientation between 30 and 60$^\circ$. Therefore, the role of the peptide terminus may be crucial in the penetration behaviour (Dennison and Phoenix 2011b; Dennison et al. 2012a). For instance, it was shown that the change in the C-terminal of the AMPs can stabilise the secondary structure and enhance the affinity of the peptide towards the membrane (Cao et al. 2005; Ali et al. 2001; Dennison et al. 2009a, 2012a; Dennison and Phoenix 2011b).

For a number of these peptides, post-translational modifications are also essential for their antimicrobial activity and the most common of these structural modifications is carboxyamidation (Sforca et al. 2004; Shalev et al. 2002; Dennison and Phoenix 2011b, b). Recently, studies by Dennison et al. have shown that aurein 2.5 (GLFDIVKKVVGAFGSL-CONH$_2$) has greater antimicrobial potency against *Klebsiella pneumonia* than its carboxylated C-terminal analogue (GLFDIVKKVVGAFGSL-COOH) (Dennison et al. 2012a).

Previous research has demonstrated that the peptide structure is crucial in the binding to target membranes. It has been proposed that the key driver for increased efficacy by amidation is due to enhanced helix stability at the membrane interface, which supports the observation that amidated peptide modelin-5-CONH_2_ exhibited a higher level of helicity in comparison to modelin-5-COOH (Dennison and Phoenix 2011b). In support of this observation other researchers such as Sforca et al. suggested that the reduced antibacterial activity of non-amidated peptides is caused not only by the decreased positive surface charge but also by the structural perturbation of the amphiphilic $\alpha$-helix, which affects its ability to disturb the cell membrane (Sforca et al. 2004). However, other researchers have shown that the N-terminal pGlu and amidated C-terminal do not significantly influence the $\beta$-hairpin amphiphilic structure of Gomesin but are critical for Gomesin's antimicrobial activity (Machado et al. 2012).

Despite the large body of experimental work on the role of amidation in the membrane interaction of AMPs using a wide range of techniques, such as Nuclear magnetic resonance (NMR) (Haney and Vogel 2009), circular dichroism (CD) (Greenfield 1999), fluorescence (Bocchinfuso et al. 2011), and lipid monolayer analysis (Dennison et al. 2010), detailed understanding of the mechanism underpinning this role is not yet fully understood. In response, molecular dynamics (MD) provide detailed information at the molecular level into many biological systems, giving an insight into the mechanism of interaction of amidated AMPs with the membrane (Cheng et al. 2011; Chen et al. 2011).

For example, Wang et al. (2012) used MD simulations to show that in the presence of a mixed 1-palmitoyl,2-oleoyl-sn-glycero-3-phosphocholine (POPC) and 1-palmitoyl-2-oleoyl-sn-glycero-3-phosphoglycerol (POPG) lipid bilayer (1:2), the C-terminus amidated CM15 peptide interacted more strongly with the bilayer than the zwitterionic POPC. Dennison and Phoenix (2011b) proposed that the mechanism for amidated peptide isoforms to interact with membranes may be similar to the mechanism for melittin pore formation proposed by van den Bogaart et al. (2008). This mechanism shows that the initial binding of peptide to the membrane competes with peptide insertion and the ability of the peptide to form pores. Irudyam et al. used a coarse-grained and atomistic MD approach to investigate the binding and reorientation of melittin to the POPC bilayer (Irudayam and Berkowitz 2012). These authors suggested that the stabilisation of the helix at the membrane interface increases the concentration of melittin bound to the membrane, enabling stable pore formation and hence the N-terminus of the peptide to reorientate. Researchers have shown that stabilising the helix at the membrane interface increases the local peptide concentration, which leads to pore formation, or carpeting of the outer leaflet, thereby causing bilayer disruption. In support of this suggestion Dos Santos Cabrera et al. (2008) undertook MD analysis on amidated and non-amidated decapeptide anoplin (ANP) in 2,2,2-trifluoroethanol (TFE)/water mixtures. The latter studies provided detailed MD information on the stability of the peptide isoforms, supporting previous findings that the amidated peptide had a more stable $\alpha$-helical conformation compared to the non-amidated isoforms (Dos Santos Cabrera et al. 2008).

Although MD has been used to gain insight into peptide/membrane interactions, an accurate, full atomistic MD investigation of the role played by amidated and non-amidated peptide is lacking. In the present work, analogues of aurein AMPs isolated from Australian Southern Bell frogs, *Litoria aurea*, were used to investigate the role of amidation in the mechanism of membrane interaction (Cheng et al. 2009). These peptides show activity against a broad range of micro-organisms (Apponyi et al. 2004) and tumour activity (Rozek et al. 2000). In this study, aurein 2.6 and 3.1 are used to investigate the role of amidation in the mechanism of membrane action with two model lipid bilayers, 1,2-dimyristoyl-sn-glycero-3-phosphocholine (DMPC) and 1,2-dimyristoyl-sn-glycero-3-phosphoserine (DMPS), which mimic the mammalian cell membrane. Generally, phosphatidylserine (PS) is found primarily in the inner leaflet while phosphatidylcholine (PC) is found in the outer leaflet. Here, MD and CD spectroscopy were used to investigate the membrane interactions of aurein peptides and these data were compared to corresponding analyses on C-terminally non-aminated isoforms of the peptide.

## Methods and materials

### Materials

1,2-Dimyristoyl-sn-glycero-3-phosphocholine (DMPC) and 1,2-dimyristoyl-sn-glycero-3-phosphoserine (DMPS) were obtained from Avanti Polar Lipids (Alabaster, AL, USA) and used without further modification. The peptide analogues of aurein peptides—aurein 2.6-COOH (GLFDIAKKVIGVIGSL-COOH), aurein 2.6-CONH$_2$ (GLFDIAKKVIGVIGSL-CONH$_2$), aurein 3.1-COOH (GLFDIVKKIAGHIAGSI-COOH), and aurein 3.1-CONH$_2$ (GLFDIVKKIAGHIAGSI-CONH$_2$)—were synthesised by SevernBiotech (UK) by solid-state synthesis and purified by HPLC to purity greater than $>$95 %. 2,2,2-Trifluoroethanol (TFE) and all other solvents and reagents were supplied by Fisher Scientific UK.

### Circular dichroism measurements

The CD spectrum was recorded on a J-815 spectropolarimeter (JASCO, UK) equipped with a Peltier temperature control unit maintained at 30 $^\circ$C as previously described (Dennison et al. 2013). All CD experiments were undertaken using a 10-mm path-length cell over a 260–180-nm wavelength range at a scan speed of 50 nm/min, 1 nm band width, and a data pitch of 0.5 nm. For all spectra acquired, ten scans per sample were performed and averaged, and the baseline acquired in the absence of peptide was subtracted. Samples were prepared by dissolving each peptide in phosphate-buffered saline (PBS, pH 7.5) and 100 % TFE to give a final peptide concentration of 0.01 mg ml$^{-1}$. CD structural analysis was also performed with aurein 2.6 and aurein 3.1 peptide isoforms in the presence of lipid. To obtain small lamellar vesicles (SUVs), a predetermined amount of dried (5 mg ml$^{-1}$) DMPC and DMPS was dissolved in chloroform, evaporated under a nitrogen stream, and placed under a vacuum overnight. The lipid film was then rehydrated using PBS (pH 7.5) and sonicated for 1 h or until the solution was no longer turbid. Liposomes were then extruded 11 times through a 0.1-$\mu$m polycarbonate filter using an Avanti polar lipid mini-extruder apparatus. Peptide/lipid samples were prepared by adding stock peptide solution to a measured volume of lipid suspension to obtain the desired peptide:lipid molar ratio (1:100) before thorough mixing. For secondary structure estimation, the percentage $\alpha$-helical content of the CD spectra was then estimated using the CDSSTR, CONTIN, and SELCON3 algorithm (protein reference set 3,4,7, SPI75 and smo 180) on the DichroWeb server (Whitmore and Wallace 2004, 2008; Whitmore et al. 2010).

### Computational method

The amidation effects of three aurein 2.6 and aurein 3.1 in different environments including water, TFE, DMPC, and DMPS were simulated by MD simulations. The mechanism of interaction between each aurein analogue and 0.1 mol/l aqueous solution, TFE, DMPC, or DMPS was examined. The aurein peptides were assembled as a canonical $\alpha$-helix using AMBER tools 1.5. All simulations were performed with the GROMACS software package (Van Der Spoel et al. 2005; Hess et al. 2008; Mura et al. 2013; Dennison et al. 2013). The GROMOS53a6 force field was used to model the peptides in the presence of TFE (Fioroni et al. 2000; Malde et al. 2011; Mura et al. 2013; Dennison et al. 2013). Water was represented by a simple point charge (SPC) (Van Der Spoel et al. 1998). The force field for DMPS and DMPC were based on GROMOS53a6 force fields taken from the literature: Poger and Mark (2010), Nesterenko and Ermakov (2013), Malde et al. (2011), and Kandt et al. (2010). All structures were equilibrated at room temperature in water (NVT and NPT simulations). Two configurations were considered: in one case the peptides were outside the lipid bilayer system with the helical axis parallel to the lipid bilayer surface while in the second case the peptides were inserted into the lipid bilayer with the helical axis parallel to the *Z* direction and relaxed in accordance with the protocol in Kandt et al. (2010). In the first case the peptides were placed at a distance of 3 nm from the top leaflet of the lipid bilayer with its axis perpendicular to the lipid-water interface (Mura et al. 2013). The simulations were performed by solvating the box with the peptides or the peptide/lipid, and counter ions Na$^+$ and CL$^-$ were added to neutralise the systems (see Table 2). Each system was equilibrated at 310 K in the sequence minimisation, NVT, and NPT simulations. An equilibration run of 2-ns NVT and 2-ns NPT was undertaken for all systems with the position of the peptide restrained using harmonic restraints with a constant force of 1.0 kJ$^{-1}$ nm$^{-2}$ per atom (Tieleman 2012; Chen et al. 2011; Kukol 2009; Mura et al. 2013). The cutoff for both van der Waals and Coulombic interactions is 1.2 nm. The particle mesh Ewald (PME) method was used in all simulations. The temperature was coupled for single groups with a constant time for the coupling of 0.1 ps to 310 K using the Berendsen thermostat. A semi-isotropic Berendsen barostat was also used with a coupling time of 2.0 ps (Piggot et al. 2011; Kukol 2009; Mura et al. 2013; Dennison et al. 2013). Simulations were undertaken at constant temperature, pressure, and number of molecules. All simulations were undertaken with constraints on all bond lengths using the LINCS algorithm (Hess et al. 1997). Then 200-ns simulations were performed in the NPT ensemble using periodic boundary conditions. The components of each system are shown in Table 1 (Dennison et al. 2013).

**Table 1:** Details of the MD simulations for aureins 2.6 (A 2.6) and aureins 3.1 (A 3.1)

	Box size (nm)	Water	Ions
Water environment			
CONH_2_			
A 2.6	8.0 × 8.0 × 8.0	16,815	31Na$^{+}$:34Cl^−^
A 3.1	8.0 × 8.0 × 8.0	16,808	31Na$^{+}$:34Cl^−^
COOH			
A 2.6	7.0 × 7.0 × 7.0	11,117	21Na$^{+}$:24Cl^−^
A 3.1	6.8 × 6.8 × 6.8	10,180	19Na$^{+}$:22Cl^−^

	Box size (nm)	TFE	Ions
TFE environment			
CONH_2_			
A 2.6	6.8 × 6.8 × 6.8	2470	21Na$^{+}$:24Cl^−^
A 3.1	6.8 × 6.8 × 6.8	2503	2Na$^{+}$:5Cl^−^
COOH			
A 2.6	7.7 × 6.8 × 6.8	1390	11Na$^{+}$:14Cl^−^
A 3.1	7.7 × 6.8 × 6.8	1368	11Na$^{+}$:14Cl^−^

	Box size (nm)	Water	Ions
DMPC 128 lipids peptide outside			
CONH_2_			
A 2.6	6.5 × 6.5 × 9.1	7478	3Cl^−^
A 3.1	6.3 × 6.3 × 9.3	7388	3Cl^−^
COOH			
A 2.6	6.3 × 6.3 × 9.3	7413	3Cl^−^
A 3.1	6.4 × 6.4 × 9.1	7416	3Cl-
DMPC 127 lipids peptide inside			
CONH_2_			
A 2.6	6.5 × 6.5 × 9.5	7981	3Na$^{+}$:6Cl^−^
A 3.1	6.3 × 6.3 × 7.1	4601	2Na$^{+}$:5Cl^−^
COOH			
A 2.6	6.4 × 6.4 × 9.0	7138	3Na$^{+}$:6Cl^−^
A3.1	6.4 × 6.4 × 7.0	4602	2Na$^{+}$:5Cl^−^
DMPS 128 lipids peptide outside			
CONH_2_			
A 2.6	5.5 × 5.5 × 12.6	8073	128Na$^{+}$:3Cl^−^
A 3.1	5.5 × 5.5 × 12.4	8066	128Na$^{+}$:3Cl^−^
COOH			
A 2.6	5.5 × 5.5 × 12.8	8083	128Na$^{+}$:3Cl^−^
A 3.1	5.5 × 5.5 × 12.3	8065	128Na$^{+}$:3Cl^−^
DMPS peptide inside			
CONH_2_			
A 2.6	5.7 × 5.7 × 8.3	4642	125Na$^{+}$:2Cl^−^
A 3.1	5.7 × 5.7 × 9.8	6346	128Na$^{+}$:3Cl^−^
COOH			
A 2.6	5.7 × 5.7 × 8.7	5076	125Na$^{+}$:2Cl^−^
A 3.1	5.8 × 5.8 × 9.3	6630	127Na$^{+}$:3Cl^−^

**Table 2:**
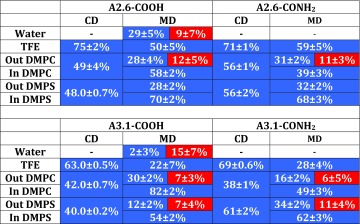
The CD and MD secondary structure of the aureins in different environments

The secondary structure in MD has been averaged over the last 50 ns of the simulations. Blue boxes ratio of $\alpha$-helix; red boxes ratio of $\beta$-sheet; the dash represents 100 % random coil

We calculated the hydrogen bonds using the post-processing ghbond option in GROMACS. The cutoff angle is 30$^\circ$ and cutoff distance 3.5 Å.

## Results

### Amidation effects on the structure and stability of aurein peptides

Amidation of the C-terminus of peptides may disturb the intramolecular hydrogen bonds (HBs) in peptides and in the intermolecular ones between peptides and the solvent molecules. Thus the driving force to form $\alpha$-helical or $\beta$-sheet structures can be varied. Previous studies have proposed that the secondary structures of AMPs are likely to be affected by amidation of the C-terminus (Dennison et al. 2013). Our investigation focuses on the peptide membrane interaction using experimental and computational techniques.

#### Secondary structure analysis using CD

**Fig. 1:**
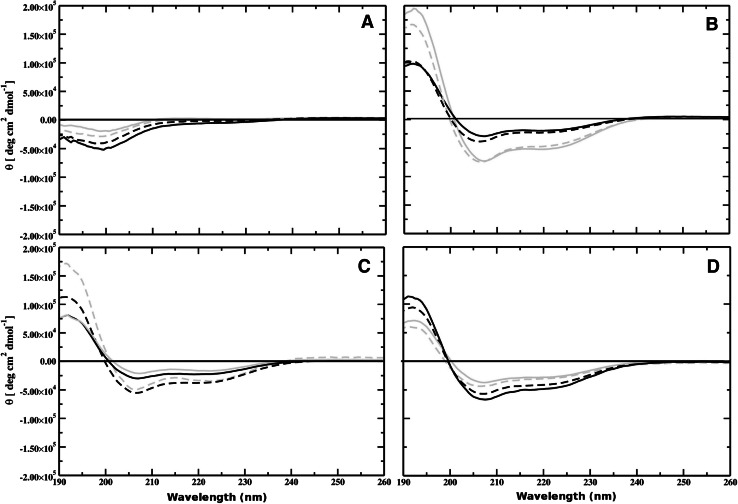
CD spectra of aurein 3.1-CONH$_2$ (*black*), aurein 3.1-COOH (*grey*), and aurein 2.6-CONH$_2$ (*dotted black*) aurein 2.6-COOH (*dotted grey*) in the presence of aqueous solution (**a**), TFE (**b**), DMPC (**c**), and DMPS (**d**)

In order to investigate the secondary structures of aurein 2.6 and aurein 3.1 isoforms in membrane mimetic environments, CD spectra were measured in PBS (pH 7.4), TFE, DMPC, and DMPS membranes (Fig. 1). Aurein 2.6 and 3.1 isoforms in PBS displayed spectral characteristics of a random coil structure (Fig. 1a). However, in a membrane environment each peptide studied adopted an $\alpha$-helical conformation displaying characteristic minima at 221–222 and 209–210 nm and a maximum at about 190 nm (Fig. 1b, c). The spectrum of aurein 2.6-COOH showed enhanced levels of helicity (75 %) in the presence of TFE compared to that of aurein 2.6-CONH$_2$ (71 %). In contrast, the spectrum of aurein 3.1-CONH$_2$ (69 %) showed an enhanced level of helicity compared to aurein 3.1-COOH (62 %), suggesting that amidation increased the propensity for the amidated peptide to form an $\alpha$-helical structure. Since it is widely accepted that cationic AMPs adopt an enhanced helical structure at a membrane-lipid interface (Dennison et al. 2013), CD experiments were undertaken in the presence of DMPC and DMPS lipid vesicles (Fig. 1c, d). CD spectroscopy spectral analysis in the presence of DMPC vesicles induced 56 % helical content in aurein 2.6-CONH$_2$ and 49 % helicity in the case of aurein 2.6-COOH, showing that amidation enhanced the helical content (Fig. 1c). However, for aurein 3.1 isoforms in the presence of DMPC vesicles CD analysis showed that both peptides were *circa* 40 % helical (Table 2), indicating that amidation had no effect on these membranes. In contrast, in the presence of anionic DMPS vesicles (Fig. 1d), the spectrum of both amidated and non-amidated aureins showed a significant increase in helical content with aurein 2.6-CONH$_2$ exhibiting a higher helical content (56 %) compared to aurein 2.6-COOH (48 %). Similarly, in the presence of DMPS vesicles aurein 3.1-CONH$_2$ exhibited a higher helical content (63 %) compared to aurein 3.1-COOH (43 %).

#### Secondary structure analysis using MD

The secondary structure was analysed according to Joosten et al. (2011) and Kabsch and Sander (1983). The average aurein conformation was calculated over the last 50 ns of simulation since fewer conformational changes on the secondary structure were obseved. Aureins in a water environment exhibited an unstable secondary structure. However, for the non-amidated peptides the percentage of $\alpha$-helix or $\beta$-sheet was maintained (see Table 2). In the presence of TFE the aureins displayed a stable $\alpha$-helical conformation, which was slightly affected by the terminal section (see Table 2). Aureins 2.6 and 3.1 showed a different $\alpha$-helical conformation in the presence of TFE due to the difference in the number of amino acids (16 for aurein 2.6 and 17 for aurein 3.1) and in the sequence of the amino acids in the peptide. Aurein 2.6 has a stable $\alpha$-helical core fomed by ALA6-LYS7-LYS8, which is more stable than the core formed by aurein 3.1 (VAL6-LYS7-LYS8), which has a mutation of the amino acid ALA6 with VAL6. In both DMPC and DMPS membranes aurein 2.6-CONH$_2$ displayed a stable $\alpha$-helical conformation (31 and 32 % respectively). In DMPC the aureins also exhibited a stable $\beta$-sheet structure (12 %). Aurein 2.6-COOH exhibited a lower percentage of $\beta$-sheet structure compared to the amidated peptide isoforms (28 % in DMPC and DMPS). The $\alpha$-helical conformation of aurein 3.1-CONH$_2$ is more stable in the presence of DMPS (24 %) than DMPC (16 %), whilst in the case of aurein 3.1-COOH the $\alpha$-helical conformation is more stable in the presence of DMPC (30 % against 12 %). For all aurein 3.1 isoforms a stable $\beta$-sheet conformation is also observed. In general peptides located in the membrane exhibited a greater percentage of $\alpha$-helical structure compared to peptides on the surface of the lipid bilayer. The highest percentage (82 %) of helical structure was observed for aurein 3.1-COOH inside the DMPC lipid bilayer (Table 2). In TFE the percentage helicity obtained computationally and experimentally showed a similar trend; however in the presence of lipid there were some differences in the values obtained.

### Dynamics properties of aureins in the presence of the lipid bilayer using MD.

#### Role of the C-terminus in the peptide/lipid bilayer interaction

**Fig. 2:**
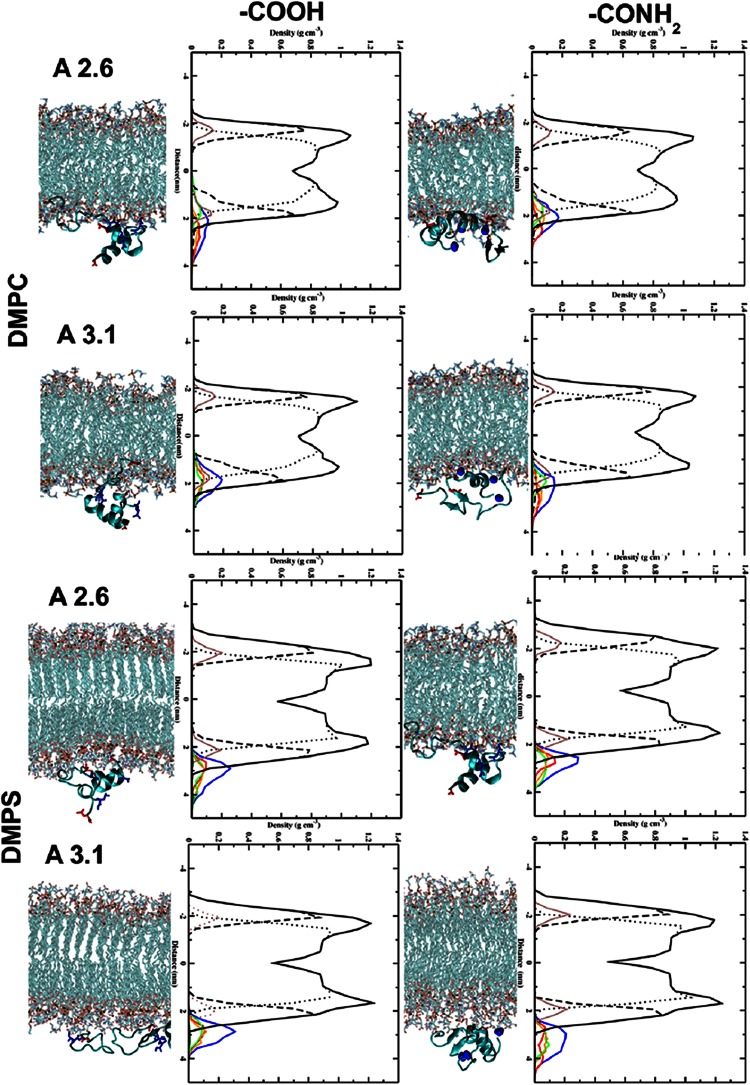
Snapshot, after 200 ns, of three aureins 2.6 and 3.1 starting outside the DMPC and DMPS membrane. The N-terminus (in *red*) and C-terminus (in *blue*) are highlighted in the graphic. The *right-hand graphics* in *each panel* show the partial densities of the components calculated in the last 50 ns of simulation: overall lipid density (*solid black line*), lipid head groups (*dots*), lipid tail groups (*dashed line*), phosphate atoms (*brown line*), the three peptides (*blue line*), peptide A (*red line*), peptide B (*green line*), and peptide C (*orange line*)

**Fig. 3:**
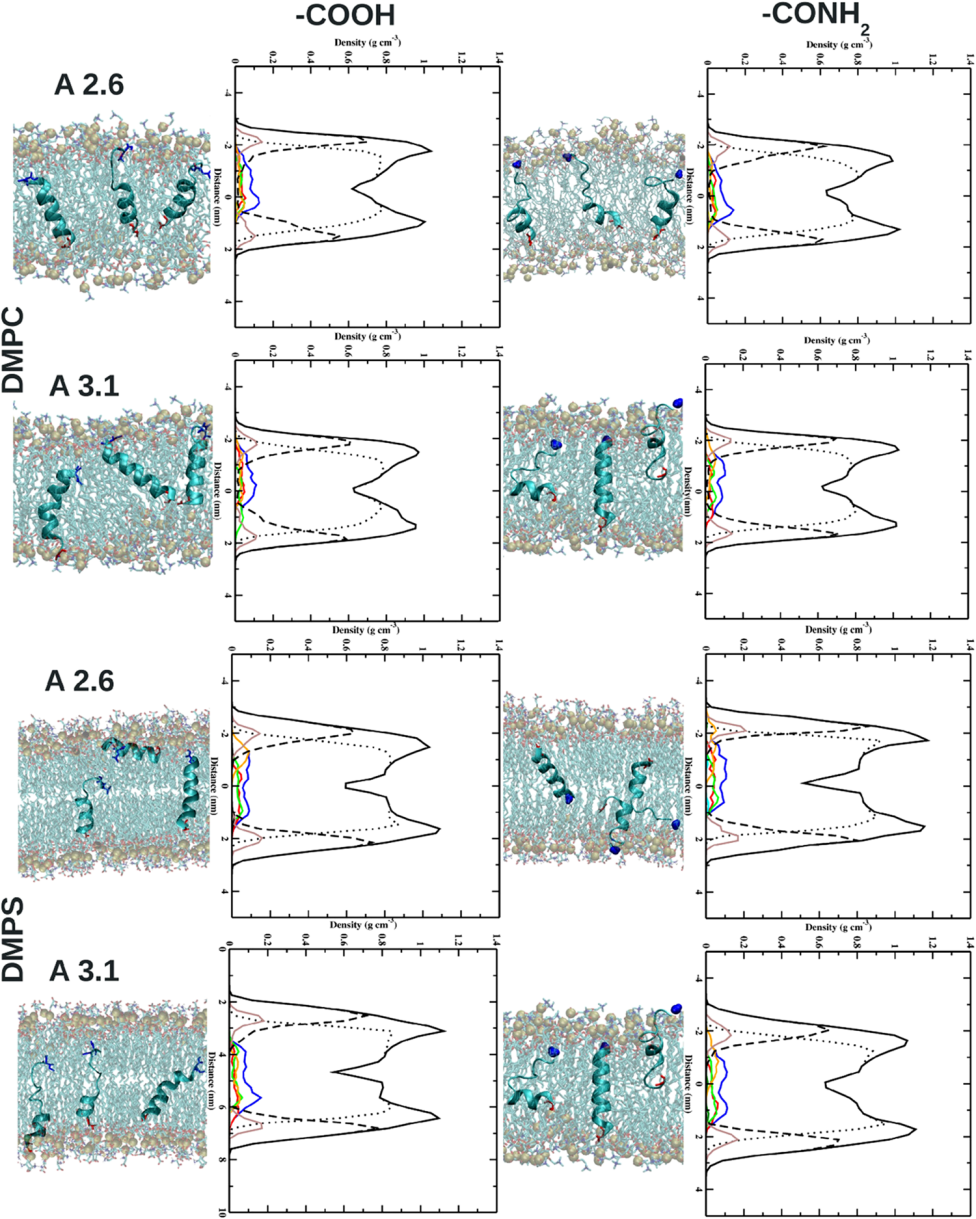
Snapshot, after 200 ns, of three aureins 2.6 (A 2.6) and 3.1 (A 3.1) starting inside the DMPC and DMPS membrane. The N-terminus (in *red*) and C-terminus (in *blue*) are highlighted in the graphic. The *right-hand graphics* in *each panel* show partial densities of the components calculated in the last 50 ns of simulation: overall lipid density (*solid black line*), lipid head groups (*dots*), lipid tail groups (*dashed line*), phosphate atoms (*brown line*), the three peptides (*blue line*), peptide A (*red line*), peptide B (*green line*), and peptide C (*orange line*)

In both peptides the presence of NH$_2$ at the C-terminus enables deeper penetration with the head groups of the DMPC lipid bilayer (Fig. 2). Here, the insertion of the N- (in red in Fig. 2) and C-terminus (in blue in Fig. 2) of the peptide is observed. In the case of non-amidated aureins the peptides interact with the lipid bilayer through the N-terminal (in red in Fig. 2) residues favouring a tilted insertion (see supplementary information). Moreover, insertion of aurein peptide into the DMPS bilayer is observed in comparison to a DMPC bilayer. In contrast, a different behaviour is observed in the case of amidation on the C-terminus. Here, the NH$_2$ group interacts with the head group of the DMPS lipid bilayer enhancing the interaction of the N- and C-terminus of the peptide with DMPS (see Fig. 2). The non-amidated aureins are situated in the water-lipid interface region with the N-terminus and interact with the head groups of the lipid bilayer. Different residues in the amidated aureins are involved in the interaction with the lipid bilayer, influencing the mechanism of penetration. The NH$_2$ at the C-terminus is influenced by the presence of PO$_4$ groups in DMPC and CO$_2$ in the DMPS lipid bilayer. In the second set of simulations the three peptides are inside the lipid bilayer. Inside the DMPC and DMPS bilayer the presence of NH$_2$ at the C-terminus highlights that each single peptide interacts with the head groups of the DMPC lipid bilayer (Fig. 3). In contrast, in the case of aurein 2.6-COOH, the C-terminus interacts with the inner part of the head groups of the DMPC lipid bilayer showing increased mobility of the peptides inside the hydrophobic core of the phospholipid bilayer (Fig. 3). The peptides after 200 ns show a very stable secondary structure inside the lipid bilayer.

#### Hydrogen bonds

**Fig. 4:**
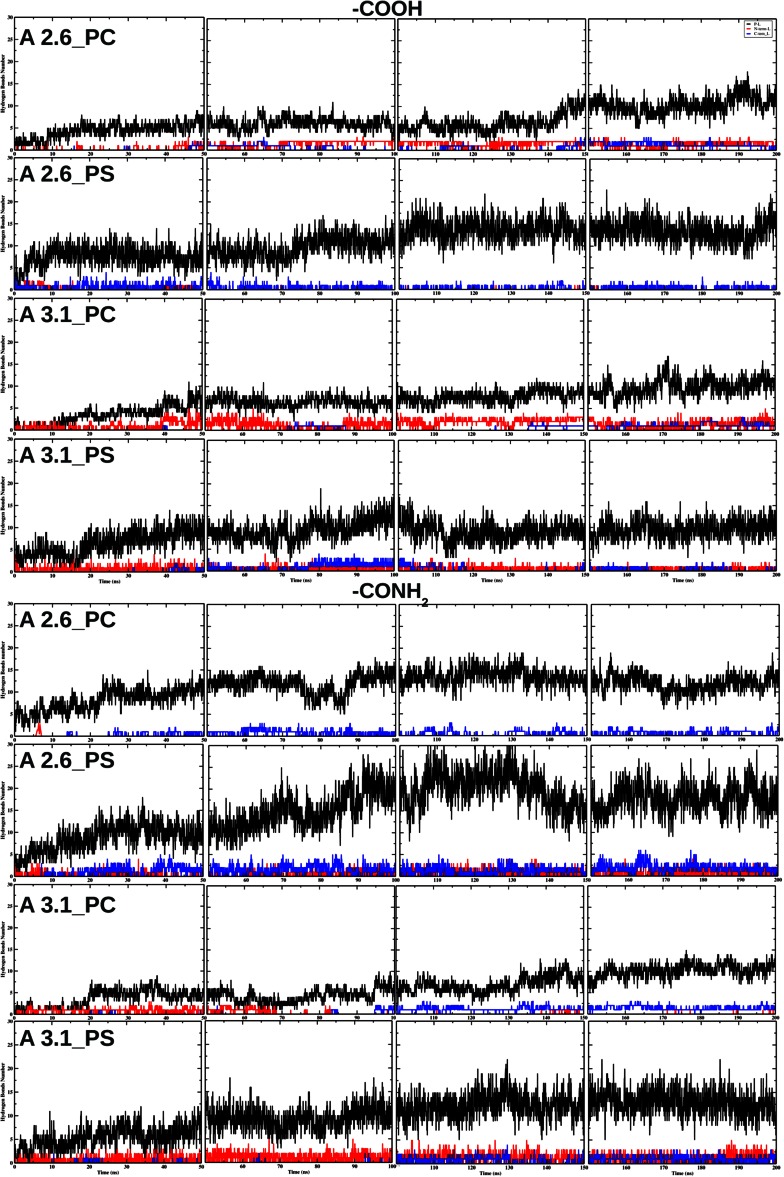
The hydrogen bond number vs. time for three aureins 2.6 and aureins 3.1 (when the peptides are starting from the configuration outside the lipid bilayer), between peptides and lipid molecules (*P*–*L*), between C-terminal groups of the peptide (COOH or CONH$_2$) and lipid bilayer (C-term-L), and between the N-terminal groups of the peptide and lipid bilayer (N-term-L)

Hydrogen bond (HB) analysis was performed for interactions between the peptide, N-terminus, or peptide C-terminus (for the aureins outside the membrane) and the head groups of the membrane as a function of the simulation time. The number of HBs between the peptide and lipid bilayer increases with time as the peptide interacts more deeply with the lipid bilayer (Fig. 4). In the majority of cases after 50 ns *circa* 10 HBs are observed between the peptides and lipid bilayer. The number of HBs between the C- and N-terminus and lipid bilayer depends on the presence of the NH$_2$ group on the C-terminus. For carboxylate peptides the number of HBs between the terminus and lipid bilayer is strongly influenced by the lipid bilayer and bonding properties of the peptide sequence. *Circa* 2.5 HBs between the N-terminus and lipid bilayer are observed for aurein 2.6-COOH in DMPC. The HBs between the C-terminus and lipid bilayer are observed after 40 ns (2 HBs) (Fig. 4). *Circa* 1 HB between the C-terminus and lipid bilayer is observed for aurein 2.6-COOH in DMPS. In contrast, HBs between the N-terminus and lipid bilayer are observed only at the beginning of the simulations (0–10 ns) and are very unstable. *Circa* 3 HBs between the N-terminus and lipid bilayer are observed for aurein 3.1-COOH in DMPC. The HBs between the C-terminus and lipid bilayer are observed after 140 ns (2 HBs) (Fig. 4). Aurein 3.1-COOH in DMPS shows a different trend compared to the previous peptides; *circa* 2 HBs between the N-terminus and lipid bilayer are observed during the simulations. While the HBs between the C-terminus and lipid bilayer are unstable with a relatively short-lived formation, *circa* 3 HBs between the C-terminus and lipid bilayer are observed after 20 ns for aurein 2.6-CONH$_2$ in DMPC, while there are no HBs between the N-terminus and lipid bilayer. Aurein 2.6-CONH$_2$ in DMPS has an increased number of HBs (*circa* 3) between the C-terminus and lipid bilayer compared to aurein 2.6-COOH. HBs between the N-terminus and lipid bilayer are also more stable during the simulation time (*circa* 2.5 HBs) (Fig. 4). *Circa* two HBs between the N-terminus and lipid bilayer are observed up to 70 ns aurein 3.1-CONH$_2$ in DMPC. The HBs between the C-terminus and lipid bilayer are observed after 95 ns (*circa* 2 HBs). *Circa* 3 HBs between the N-terminus and lipid bilayer are observed during the simulations for aurein 3.1-CONH$_2$ in DMPS. While the HBs between the C-terminus and lipid bilayer (*circa* 2) are observed after 100 ns, there is no significant difference in the formation of HBs between the peptides and lipid bilayer. The presence of NH$_2$ at the terminus of the peptide showed a larger number of HBs between the peptide C-terminus and lipid bilayer (red line in Fig. 4).

## Discussion

A wide range of structural conformations has been displayed by AMPs (Maget-Dana 1999) and the ability of an AMP to interact with a target membrane not only depends upon the peptide structural characteristics but also the membrane target (Nguyen et al. 2011). The mechanism of AMP interaction is dependent on the membrane environment and currently no single model can explain the mechanism of action. However, it is generally accepted that the targeting and binding of the peptide to the membrane are via electrostatic interactions between the cationic regions of the peptide and negatively charged moieties occurring in membranes of these target cells (Dennison et al. 2007). Once associated with the membrane, one factor that has been identified as important for activity is the stability of the secondary structure prior to membrane penetration (Giangaspero et al. 2001; Dennison et al. 2012b, a). Cabrera et al. stated that C-terminal amidation is important for both the helix stability and lytic activity of peptides (dos Santos Cabrera et al. 2004). CD and MD structural studies indicated that both aurein isoforms were unstructured in solution (Fig. 1; Table 2) and then fold to form an $\alpha$-helical conformation in the presence of a lipid interface (Dennison et al. 2013).

Many studies have shown that the antimicrobial activity of AMPs is related to the positive charge of peptides (Giangaspero et al. 2001; Dennison et al. 2009b, 2003, 2005; Benko-Iseppon et al. 2010). Amidated peptides have a higher positive charge than the corresponding non-amidated peptide, which may be the reason why some are more active. The greater net charge on aurein 2.6-CONH$_2$ and aurein 3.1-CONH$_2$ compared to aurein 2.6-COOH and aurein 3.1-COOH would therefore be predicted to enhance the membrane association. To obtain further information on the mode of action of aurein 2.6 and aurein 3.1 analogues, CD experiments were performed in the presence of TFE. Aurein 2.6-CONH$_2$ and aurein 3.1-CONH$_2$ were seen to adopt the $\alpha$-helical structure in the presence of TFE [71 and 61 % $\alpha$-helix respectively (Table 2)] and show a greater  helicity percentage compared to the carboxyl-free C-terminus. The same behaviour has been confirmed by MD simulations, which showed that the peptides exhibited a stable conformation (Table 2). In the presence of lipids CD analysis showed that the aurein 2.6-CONH$_2$ possesses a greater helicity compared to aurein 2.6-COOH supported by MD analysis. Furthermore, in DMPC, the aurein 2.6 can bind the stabilising helix at the membrane interface (Table 2), which leads to different modes of interaction and membrane binding compared to aurein 3.1.

Peptide binding and partitioning into a membrane are affected by the ability of an AMP to alter the lipid polymorphism of a membrane (Epand 1997). A number of factors determine the lipid polymorphism of a membrane, for example the location of the peptide in a membrane, the presence of amino acids, which contain several C-C bonds in the side chain such as LYS, and physiochemical characteristics such as charge and amphiphilicity, with evidence to show that hydrophobicity determines the level of membrane partitioning. The MD analysis in Fig. 2 shows that for aurein 2.6-COOH and aurein 3.1-COOH in the presence of a DMPC and DMPS bilayer, the peptides orientate horizontally to the water-lipid interface, allowing shallow penetration into the hydrophobic core of the lipid interior. Here the LYS amino acids, which are centrally located in the peptide, are interacting with the head groups of the lipid bilayer forming HBs with the lipid bilayer. This horizontal orientation of the peptide would lead to lipid head groups being pushed aside by the peptide and hence forcing a gap in the membrane hydrophobic region. This result is comparable to other AMPs, which may utilise a carpet or toroidal pore-type mechanism (Sato and Feix 2006; Hoskin and Ramamoorthy 2008; Fernandez et al. 2012). The amidated C-terminus of the peptide can also form a larger number of HBs in comparison with carboxyl peptides in all cases. This affects the secondary structures and activities of the amidated and non-amidated aurein 2.6 and aurein 3.1 at the water-lipid interface.

The amidated peptides tilt their helices (Fig. 2) and hence the lipids distort their acyl chains by compressing the lipids to match the peptide molecular architecture or by stretching of the acyl chains (Ramadurai et al. 2010). Ramadurai et al. showed that the degree of the tilt is due to positive and negative mismatch due to the presence of snorkelling Lys residues. In turn this leads to a curvature around the peptide in the bilayer leading to membrane destabilisation (Ramadurai et al. 2010). This oblique orientation mechanism has been proposed for other aurein amidated peptides, such as aurein 1.2 (Marcotte et al. 2003) and aurein 2.3 (Mura et al. 2013), which have been shown to insert into the membrane with a shallow angle between 30$^\circ$ and 60$^\circ$ leading to membrane destabilisation.

In conclusion, our analysis highlights how the mechanism of interaction between the peptide and lipid bilayer is dependent on whether the peptide is amidated at the C-terminus. The configuration of the peptide inside the lipid bilayer also throws light on the interaction between the peptide and bilayer component. When the peptides and the lipid bilayer are in close contact, HBs are formed between the carboxyl terminal and head groups of lipid molecules, which contribute to the different behaviour of peptides at the water-lipid interface.

**Fig. 5:**
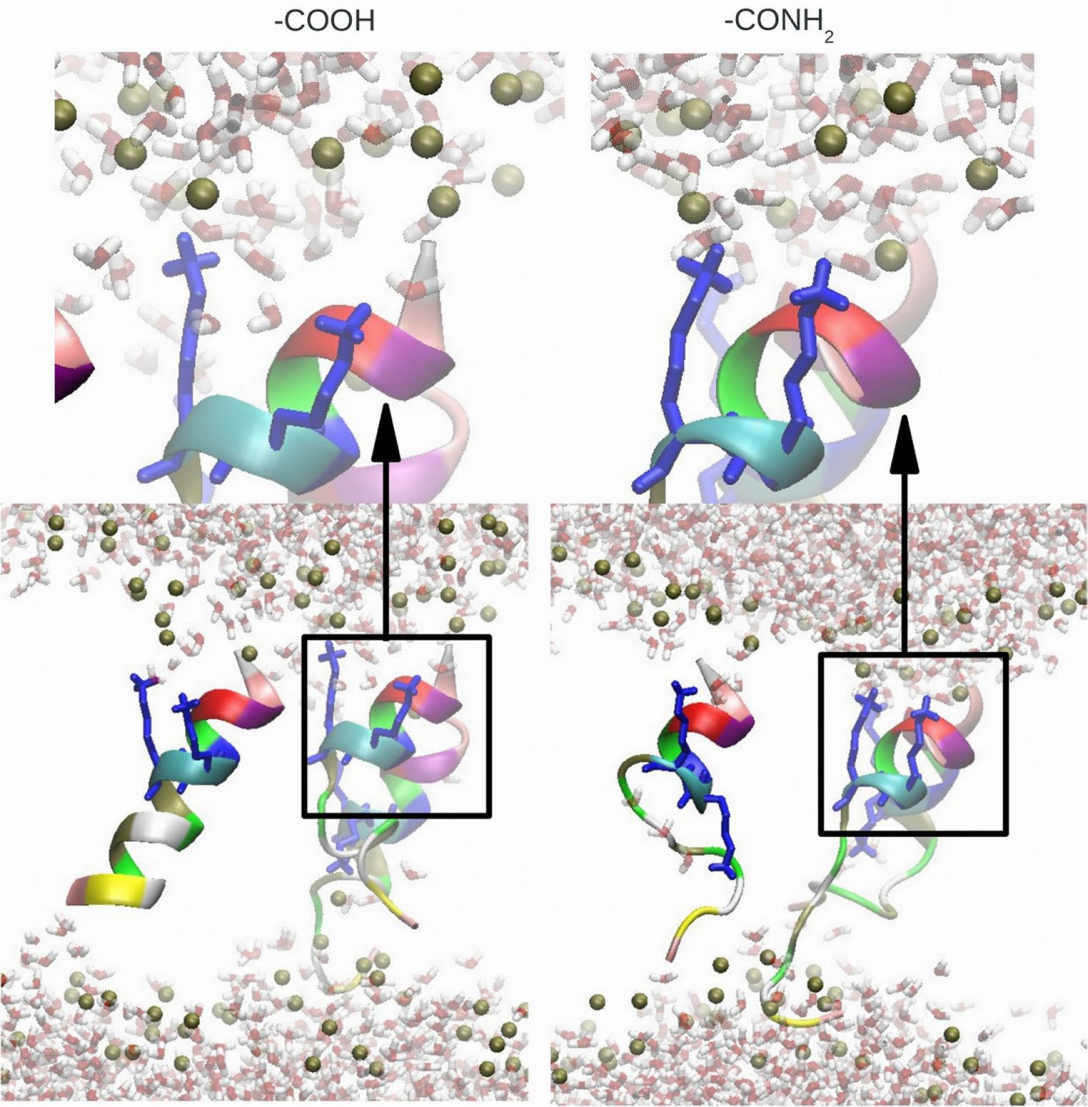
*Bottom* Interaction of the lysine residue (in *blue*) of aurein 2.6 with the phosphate atoms (*brown*) of the DMPC lipid bilayer at 200 ns. *Top* Magnified image of one $\alpha$-helical structure

Aurein 2.6-CONH$_2$ shows a contribution of $\alpha$-helical conformation larger than aurein 2.6-COOH in both the membranes while aurein 3.1-CONH$_2$ shows a larger $\alpha$-helical conformation only in the presence of the DMPC membrane. These results are in agreement with CD experiments. When the peptides are inside the membrane, the LYS interacts with the head group of the lipid bilayer, staying anchored to the hydrophilic interface region (Fig. 5). This behaviour of the LYS residue creates a perturbation on the membrane allowing the penetration of water molecules.

## Electronic supplementary material

Supplementary material 1 (PDF 4818 kb)
