# Supplementary material for: The effect of amidation on the behaviour of antimicrobial peptides
Source: Eur Biophys J. 2016 Jan 8;45:195–207. doi: 10.1007/s00249-015-1094-x (PMC4796345; doi:10.1007/s00249-015-1094-x)
Supplement: Supplementary file 1 — Supplementary material 1 (PDF 4818 kb) [file 249_2015_1094_MOESM1_ESM.pdf]

Noname manuscript No.
(will be inserted by the editor)

The effect of amidation on the behaviour of antimicrobial peptides

Manuela Mura*[2] and Jianping Wang[1] · Yuhua Zhou[1] · Marco Pinna[2] · Andrei V. Zvelindovsky[2] · Sarah R. Dennison[3,4] · David A. Phoenix[4]

December 1, 2015

[1] UCLan Biomedical Technology Limited (Shenzhen), Shenzhen Virtual University Park,Shenzhen,518057 P. R. China · [2]Computational Physics Group, School of Mathematics and Physics,University of Lincoln, Brayford Pool, Lincoln, LN6 7TS · [3]School of Pharmacy and Biomedical Science, University of Central Lancashire, Preston PR1 2HE, UK · [4]School of Applied Science, London South Bank University, 103 Borough Road, London SE1 0AA, UK

Peptide	Method	Reference set	α1	α2	Total	Peptide	Method	Reference set	α1	α2	Total
		3.00	37.00	12.00	49.00			3.00	36.00	19.00	55.00
		4.00	29.00	19.00	48.00			4.00	39.00	19.00	58.00
	CDSSTR	7.00	24.00	23.00	47.00		CDSSTR	7.00	36.00	24.00	60.00
		SP175	30.00	17.00	47.00			SP175	36.00	19.00	55.00
		SMP180	30.00	17.00	47.00			SMP180	32.00	8.00	40.00
		3.00	33.50	17.00	50.50			3.00	33.50	16.50	50.00
		4.00	30.10	16.90	47.00			4.00	33.50	26.50	60.00
Aurein 2.6-COOH DMPC	CONTIN	7.00	30.90	14.60	45.50	Aurein 2.6-CONH DMPC	CONTIN	7.00	33.50	26.50	60.00
		SP175	28.10	25.50	53.60			SP175	38.90	21.80	60.70
		SMP180	28.70	20.40	49.10			SMP180	33.70	16.40	50.10
		3.00	20.00	24.00	44.00			3.00	43.20	12.60	55.80
		4.00	20.80	27.80	48.60			4.00	49.30	13.00	62.30
	SELCON3	7.00	29.80	18.90	48.70		SELCON3	7.00	47.90	12.70	60.60
		SP175	27.30	18.50	45.80			SP175	41.10	11.40	52.50
		SMP180	26.70	14.10	40.80			SMP180	49.70	11.80	61.50
				av	47.44					av	56.10
				SD	2.91					sd	6.01
		3.00	44.00	4.00	48.00			3.00	36.00	20.00	56.00
		4.00	34.00	11.00	45.00			4.00	40.00	18.00	58.00
	CDSSTR	7.00	36.00	11.00	47.00		CDSSTR	7.00	41.00	15.00	56.00
		SP175	36.00	9.00	45.00			SP175	41.00	14.00	55.00
		SMP180	39.00	19.00	58.00			SMP180	29.00	15.00	44.00
		3.00	31.30	12.80	44.10			3.00	47.20	13.00	60.20
		4.00	32.00	13.00	45.00			4.00	47.20	13.00	60.20
Aurein 2.6-COOH DMPS	CONTIN	7.00	32.30	11.60	43.90	Aurein 2.6-CONH DMPS	CONTIN	7.00	39.20	12.30	51.50
		SP175	32.80	11.80	44.60			SP175	29.10	13.00	42.10
		SMP180	36.80	13.60	50.40			SMP180	29.00	31.00	60.00
		3.00	31.50	14.10	45.60			3.00	47.00	21.80	68.80
		4.00	31.90	15.30	47.20			4.00	40.30	21.60	61.90
	SELCON3	7.00	31.20	14.10	45.30		SELCON3	7.00	43.00	16.70	59.70
		SP175	28.60	13.00	41.60			SP175	48.60	14.50	63.10
		SMP180	26.90	12.70	39.60			SMP180	48.60	14.50	63.10
				av	46.02					av	57.31
				SD	4.17					sd	7.08

Fig. 1 The CD secondary structure of aureins 2.6 in Water and TFE fitted using CDSSTR, CONTIN and SELCON3.

Peptide	Method	Reference set	α1	α2	Total	Peptide	Method	Reference set	α1	α2	Total
Aurein 2.6-COOH water	CDSSTR	3.00	4.30	9.10	13.40	Aurein 2.6-CONH water	CDSSTR	3.00	4.30	9.10	13.40
		4.00	5.50	11.20	16.70			4.00	0.70	0.50	1.20
		7.00	1.80	1.00	2.80			7.00	0.18	0.18	0.36
		SP175	0.00	7.60	7.60			SP175	0.00	7.60	7.60
		SMP180	0.50	0.39	0.89			SMP180	0.00	0.86	0.86
	CONTIN	3.00	0.00	8.50	8.50		CONTIN	3.00	0.00	0.98	0.98
		4.00	0.20	7.00	7.20			4.00	0.00	8.70	8.70
		7.00	0.30	1.40	1.70			7.00	0.00	5.90	5.90
		SP175	0.20	0.50	0.70			SP175	0.00	10.20	10.20
		SMP180	0.00	0.79	0.79			SMP180	1.90	6.70	8.60
	SELCON3	3.00	0.10	0.20	0.30		SELCON3	3.00	4.00	3.00	7.00
		4.00	1.00	8.00	9.00			4.00	-1.00	11.00	10.00
		7.00	1.00	2.00	3.00			7.00	0.00	2.00	2.00
		SP175	0.00	0.00	0.00			SP175	1.00	3.00	4.00
		SMP180	0.00	0.00	0.00			SMP180	0.00	1.00	1.00
				av	4.84					av	5.45
				SD	5.29					sd	4.25
Aurein 2.6-COOH TFE	CDSSTR	3.00	57.00	17.00	74.00	Aurein 2.6-CONH TFE	CDSSTR	3.00	51.00	20.00	71.00
		4.00	61.00	14.00	75.00			4.00	61.00	16.00	77.00
		7.00	56.00	16.00	72.00			7.00	50.00	21.00	71.00
		SP175	61.00	17.00	78.00			SP175	60.00	17.00	77.00
		SMP180	65.00	10.00	75.00			SMP180	50.00	19.00	69.00
	CONTIN	3.00	47.00	26.60	73.60		CONTIN	3.00	53.35	27.60	80.95
		4.00	54.70	23.30	78.00			4.00	53.60	28.40	82.00
		7.00	46.10	28.90	75.00			7.00	52.90	28.40	81.30
		SP175	46.10	28.90	75.00			SP175	54.10	29.50	83.60
		SMP180	46.10	28.90	75.00			SMP180	54.10	29.50	83.60
	SELCON3	3.00	54.00	61.00	115.00		SELCON3	3.00	48.40	24.80	73.20
		4.00	52.60	24.50	77.10			4.00	45.80	23.10	68.90
		7.00	52.60	24.50	77.10			7.00	45.80	23.10	68.90
		SP175	55.00	25.00	80.00			SP175	33.00	20.50	53.50
		SMP180	45.40	22.30	67.70			SMP180	47.90	13.70	61.60
				av	77.83					av	73.50
				SD	10.68					sd	8.59

Fig. 2 The CD secondary structure of aureins3.1 in Water and TFE fitted using CDSSTR, CONTIN and SELCON3.

Peptide	Method	Reference set	α1	α2	Total	Peptide	Method	Reference set	α1	α2	Total
Aurein 2.6-COOH DMPC	CDSSTR	3.00	37.00	12.00	49.00	Aurein 2.6-CONH DMPC	CDSSTR	3.00	36.00	19.00	55.00
		4.00	29.00	19.00	48.00			4.00	39.00	19.00	58.00
		7.00	24.00	23.00	47.00			7.00	36.00	24.00	60.00
		SP175	30.00	17.00	47.00			SP175	36.00	19.00	55.00
		SMP180	30.00	17.00	47.00			SMP180	32.00	8.00	40.00
	CONTIN	3.00	33.50	17.00	50.50		CONTIN	3.00	33.50	16.50	50.00
		4.00	30.10	16.90	47.00			4.00	33.50	26.50	60.00
		7.00	30.90	14.60	45.50			7.00	33.50	26.50	60.00
		SP175	28.10	25.50	53.60			SP175	38.90	21.80	60.70
		SMP180	28.70	20.40	49.10			SMP180	33.70	16.40	50.10
	SELCON3	3.00	20.00	24.00	44.00		SELCON3	3.00	43.20	12.60	55.80
		4.00	20.80	27.80	48.60			4.00	49.30	13.00	62.30
		7.00	29.80	18.90	48.70			7.00	47.90	12.70	60.60
		SP175	27.30	18.50	45.80			SP175	41.10	11.40	52.50
		SMP180	26.70	14.10	40.80			SMP180	49.70	11.80	61.50
				av	47.44					av	56.10
				SD	2.91					sd	6.01
Aurein 2.6-COOH DMPS	CDSSTR	3.00	44.00	4.00	48.00	Aurein 2.6-CONH DMPS	CDSSTR	3.00	36.00	20.00	56.00
		4.00	34.00	11.00	45.00			4.00	40.00	18.00	58.00
		7.00	36.00	11.00	47.00			7.00	41.00	15.00	56.00
		SP175	36.00	9.00	45.00			SP175	41.00	14.00	55.00
		SMP180	39.00	19.00	58.00			SMP180	29.00	15.00	44.00
	CONTIN	3.00	31.30	12.80	44.10		CONTIN	3.00	47.20	13.00	60.20
		4.00	32.00	13.00	45.00			4.00	47.20	13.00	60.20
		7.00	32.30	11.60	43.90			7.00	39.20	12.30	51.50
		SP175	32.80	11.80	44.60			SP175	29.10	13.00	42.10
		SMP180	36.80	13.60	50.40			SMP180	29.00	31.00	60.00
	SELCON3	3.00	31.50	14.10	45.60		SELCON3	3.00	47.00	21.80	68.80
		4.00	31.90	15.30	47.20			4.00	40.30	21.60	61.90
		7.00	31.20	14.10	45.30			7.00	43.00	16.70	59.70
		SP175	28.60	13.00	41.60			SP175	48.60	14.50	63.10
		SMP180	26.90	12.70	39.60			SMP180	48.60	14.50	63.10
				av	46.02					av	57.31
				SD	4.17					sd	7.08

Fig. 3 The CD secondary structure of aureins 2.6 in different lipid environment (DMPC and DMPS) fitted using CDSSTR, CONTIN and SELCON3.

Peptide	Method	Reference set	α1	α2	Total	Peptide	Method	Reference set	α1	α2	Total
		3.00	34.00	7.00	41.00			3.00	23.00	16.00	39.00
		4.00	35.00	12.00	47.00			4.00	21.00	17.00	38.00
	CDSSTR	7.00	35.00	13.00	48.00		CDSSTR	7.00	21.00	18.00	39.00
		SP175	32.00	11.00	43.00			SP175	21.00	18.00	39.00
		SMP180	37.00	10.00	47.00			SMP180	21.00	18.00	39.00
		3.00	34.00	7.00	41.00			3.00	25.50	17.70	43.20
Aurein 3.1-		4.00	35.00	12.00	47.00	Aurein3.1-		4.00	21.70	21.20	42.90
COOH	CONTIN	7.00	35.00	13.00	48.00	CONH	CONTIN	7.00	24.50	12.40	36.90
DMPC		SP175	22.00	11.00	33.00	DMPC		SP175	26.90	18.20	45.10
		SMP180	27.00	11.00	38.00			SMP180	26.90	18.20	45.10
		3.00	28.00	16.20	44.20			3.00	28.50	19.50	48.00
		4.00	28.50	17.30	45.80			4.00	28.80	19.80	48.60
	SELCON3	7.00	28.40	17.20	45.60		SELCON3	7.00	24.20	18.60	42.80
		SP175	21.80	17.20	39.00			SP175	24.20	18.60	42.80
		SMP180	25.70	12.70	38.40			SMP180	24.00	16.00	40.00
				av	43.07					av	41.96
				SD	4.51					SD	3.63
		3.00	29.00	11.00	40.00			3.00	42.00	19.00	61.00
		4.00	27.00	16.00	43.00			4.00	44.00	18.00	62.00
	CDSSTR	7.00	28.00	15.00	43.00		CDSSTR	7.00	43.00	19.00	62.00
		SP175	22.00	21.00	43.00			SP175	43.00	18.00	61.00
		SMP180	25.00	24.00	49.00			SMP180	43.00	18.00	61.00
		3.00	31.80	19.70	51.50			3.00	56.50	12.00	68.50
Aurein 3.1-		4.00	28.20	17.70	45.90	Aurein 3.1-		4.00	56.40	13.70	70.10
COOH	CONTIN	7.00	28.70	16.50	45.20	CONH	CONTIN	7.00	56.20	12.70	68.90
DMPS		SP175	25.40	16.20	41.60	DMPS		SP175	54.40	12.40	66.80
		SMP180	25.10	14.20	39.30			SMP180	55.20	0.99	56.19
		3.00	22.40	23.00	45.40			3.00	41.50	18.40	59.90
		4.00	22.40	23.00	45.40			4.00	48.00	17.60	65.60
	SELCON3	7.00	23.90	23.60	47.50		SELCON3	7.00	47.90	17.40	65.30
		SP175	11.60	20.00	31.60			SP175	43.60	17.30	60.90
		SMP180	11.60	20.00	31.60			SMP180	47.20	15.20	62.40
				av	42.87					av	63.44
				SD	5.59					SD	3.91

Fig. 4 The CD secondary structure of aureins 2.6 in different lipid environment (DMPC and DMPS) fitted using CDSSTR, CONTIN and SELCON3.

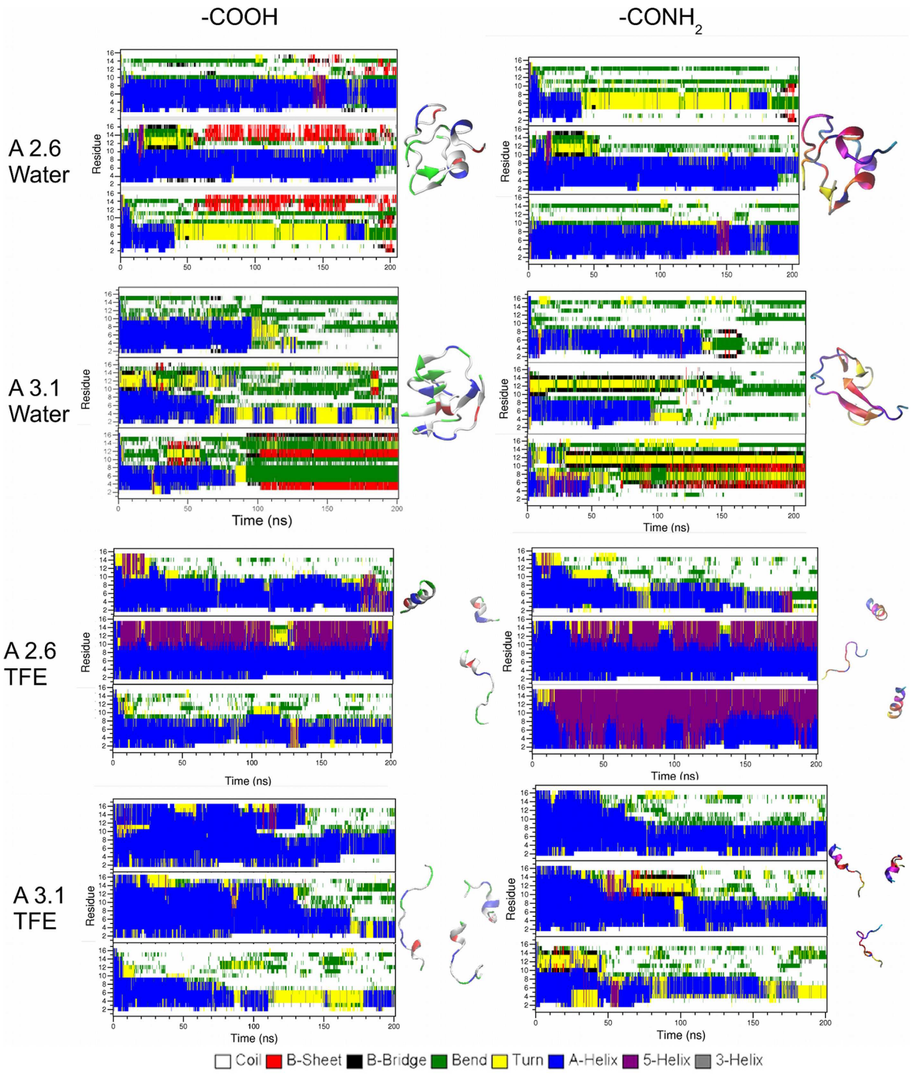

Fig. 5 Time evolution of the secondary structure of the peptides in presence of solution during the 200ns.

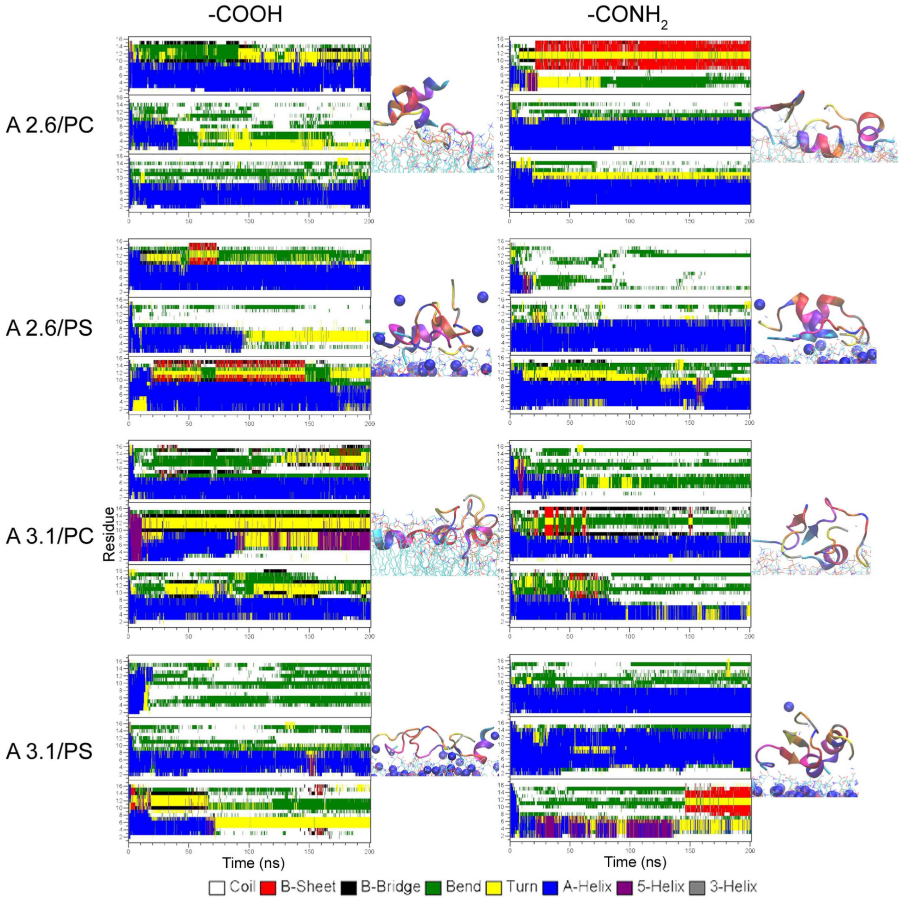

Fig. 6 Time evolution of the secondary structure of the peptides outside the lipid bilayer during the 200ns.

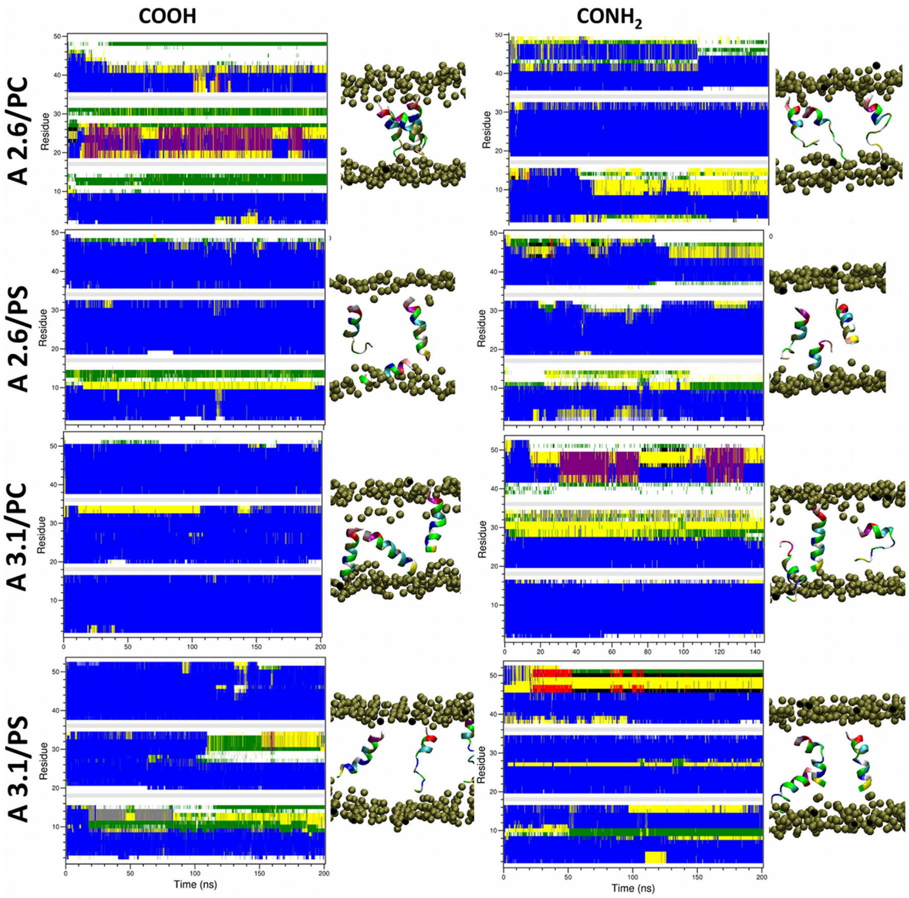

Fig. 7 Time evolution of the secondary structure of the peptides inside the lipid bilayer during the 200ns.

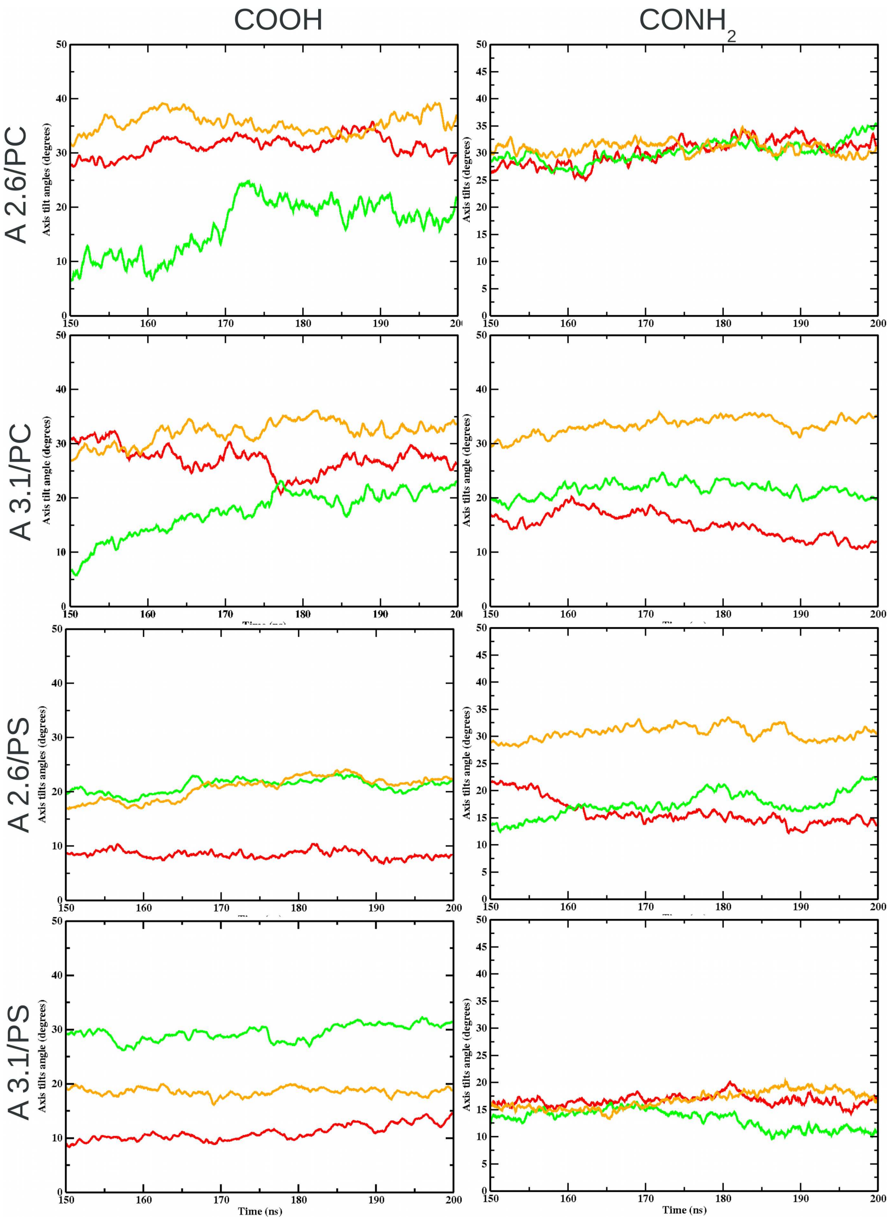

Fig. 8 Profile of the behaviour of angle between the long axis for the peptide A (red line), peptide B (green line), peptide C (orange line) of the aurein 2.6 and aurein 3.1 and the bilayer surface (DMPC and DMPS).
